# Effect of Theracurmin Products for Alleviating Alcohol Hangovers in Healthy Adults

**DOI:** 10.3390/jcm14196996

**Published:** 2025-10-02

**Authors:** Yeongtaek Hwang, Paul Kim, Minji Kwon, Sung-Vin Yim, Bo-Hyung Kim, Hyunjung Lim

**Affiliations:** 1 Department of Medical Nutrition, Graduate School of East-West Medical Science, Kyung Hee University, Yongin 17104, Republic of Korea; hyt7622@khu.ac.kr (Y.H.);; 2 Research Institute of Medical Nutrition, Kyung Hee University, Seoul 02447, Republic of Korea; 3 Department of Clinical Pharmacology and Therapeutics, Kyung Hee University College of Medicine, Kyung Hee University Hospital, Seoul 02447, Republic of Korea; 4 East-West Medical Research Institute, Kyung Hee University, Seoul 02447, Republic of Korea; 5 Department of Biomedical Science and Technology, Graduate School, Kyung Hee University, Seoul 02447, Republic of Korea

**Keywords:** hangover, alcohol, acetaldehyde, theracurmin, metabolism, clinical trial

## Abstract

**Background/Objectives:** Excessive alcohol consumption leads to hangovers, which cause discomfort and reduce work efficiency, resulting in socioeconomic losses. Theracurmin, known for its antioxidant and hepatoprotective properties, may help mitigate these effects. We evaluated the efficacy and safety of two Theracurmin-based products in alleviating hangover symptoms in humans. **Methods:** A randomized, double-blind, placebo-controlled, crossover trial was conducted in 27 healthy adults, with a balanced distribution of men and women. Two formulations were tested: Ready Q, containing Theracurmin, *Hovenia dulcis* Thunb. extract powder, and L-glutathione yeast extract; and Theracurmin, containing only Theracurmin. The products were administered on designated visit days, followed by an alcohol challenge 30 min after administration. Blood and breath alcohol profiles were assessed 15 h post-consumption, and participants completed a hangover symptom questionnaire. **Results:** Compared to placebo, Ready Q resulted in a significantly lower area under the curve (AUC) for serum alcohol concentration (−94.92 mg·h/dL [−170.91, −18.93]), as well as lower AUC (−8.441 mg·h/dL [−11.713, −5.169]) for serum acetaldehyde. Theracurmin showed similar effects, with reduced AUC (−117.21 mg·h/dL [−194.20, −40.22]) for serum alcohol concentration, and lower AUC (−8.161 mg·h/dL [−12.597, −3.725]) for corrected serum acetaldehyde levels. **Conclusions:** These findings suggest that both products effectively enhance alcohol metabolism in healthy adults, underscoring their potential as interventions for alleviating alcohol hangovers.

## 1. Introduction

In modern society, economic growth and industrialization have facilitated the expansion of culinary culture, leisure activities, and social interactions, leading to increased access to alcohol [1]. As a result, alcohol consumption has become more prevalent, contributing to the rising incidence of hangovers and alcohol-related diseases [1]. Alcohol is the most widely consumed psychoactive and toxic substance, with global per capita consumption reported at 5.5 L per year in 2019 [2,3]. On average, men consumed 8.7 L per year, compared with 2.2 L for women [3]. Alcohol is primarily metabolized in the liver through two main pathways. The major pathway involves alcohol dehydrogenase (ADH) and aldehyde dehydrogenase (ALDH), while the secondary pathway is the microsomal ethanol oxidizing system (MEOS), which becomes more active under chronic or excessive alcohol intake [4]. Metabolites such as acetaldehyde and reactive oxygen species induce oxidative stress by interacting with proteins, lipids, and DNA, resulting in cytotoxicity [5]. Persistent oxidative stress not only impairs hepatic function but also contributes to hangover symptoms through the accumulation of toxic metabolites [5]. The alcohol hangover is defined as “the combination of negative mental and physical symptoms which can be experienced after a single episode of alcohol consumption, starting when blood alcohol concentration approaches zero” [6]. Hangovers are complex conditions characterized by fatigue, headache, sensitivity to light and sound, muscle pain, thirst, dizziness, low mood, anxiety, and irritability [7]. These symptoms extend beyond discomfort and impaired individual performance; reductions in working memory contribute to decreased productivity and increased error rates in daily life. Moreover, hangovers significantly raise the risk of accidents, leading to considerable economic losses [8]. Due to these concerns, numerous traditional remedies have been used to alleviate hangover symptoms and shorten their duration; however, few have undergone rigorous scientific evaluation [7]. This underscores the need for effective, evidence-based treatments that can reliably relieve hangover symptoms, improve daily functioning and quality of life, and mitigate their economic burden.

Several studies have investigated the efficacy of herbs and fruits, such as *Hovenia dulcis*, red ginseng, and Korean pear, which are recognized for alleviating hangover symptoms [9,10]. These components are known to stimulate alcohol-metabolizing enzymes, such as ADH and ALDH, and possess antioxidant and hepatoprotective properties that may contribute to reducing alcohol toxicity and hangover severity [11,12,13]. Recent studies have also focused on developing comprehensive hangover remedies incorporating ALDH to enhance alcohol metabolism and reduce acetaldehyde toxicity [14]. Curcumin, the active component of turmeric and a widely used ingredient in curry, has demonstrated antioxidant and hepatoprotective effects [15,16]. However, its limited oral bioavailability significantly constrains its effectiveness [16,17]. To address this issue, a formulation known as Theracurmin was developed to improve curcumin solubility and increase its intestinal absorption by approximately 42-fold compared to submicron curcumin powder [16]. In this study, we evaluated the efficacy and safety of two Theracurmin-based formulations in relieving hangover symptoms in human participants—one containing Theracurmin, *Hovenia dulcis* Thunb, and L-glutathione yeast extract (HD-RQ-1405, hereafter referred to as “Ready Q”), and the other comprising Theracurmin alone (HD-RQ-2307, hereafter referred to as “Theracurmin”)—by comparing each separately with placebo (Aim I: Ready Q vs. placebo; Aim II: Theracurmin vs. placebo).

## 2. Materials and Methods

### 2.1. Study Design

A randomized, double-blind, placebo-controlled, crossover clinical trial was conducted to assess efficacy in healthy adults. Eligible participants were assigned to three products in sequence: Ready Q (containing 166.68 mg of Theracurmin CR-033P, including 50 mg of curcumin; 500 mg of *Hovenia dulcis* Thunb. extract powder; and 100 mg of L-glutathione yeast extract standardized to 50%) on the first visit, placebo on the second, and Theracurmin (containing 333.342 mg of Theracurmin CR-033P, including 100 mg of curcumin) on the third. These products used in the trial were provided by HANDOK Inc. (Seoul, Republic of Korea). Participants were instructed to avoid excessive alcohol intake and to abstain from drinking alcohol starting 2 days before each visit. Participants arrived at the research facility in the afternoon, and breath alcohol concentration was measured. If the value exceeded 0.000%, the participant was excluded from the study. Vital signs were recorded, and a low-fat standard meal was provided. Two hours after the meal, the investigational product assigned for that visit was then administered. After 30 min, participants consumed 500 mL of Soju (16.5% alcohol), containing 65.13 g of alcohol (0.95 g/kg body weight), along with 50 g of shrimp snacks (250 kcal) over 30 min. A trained clinical research coordinator collected blood samples through a catheter inserted into an antecubital vein before alcohol intake and at 0 min, 15 min, 30 min, 1 h, 2 h, 4 h, 6 h, and 15 h (after bedtime) to assess serum alcohol and acetaldehyde concentrations. Breath alcohol concentration was measured at the same nine time points. Participants completed the hangover symptom questionnaire, and safety was assessed at 15 h by evaluating vital signs (blood pressure, pulse rate, temperature) and conducting laboratory tests. Participants were advised to maintain consistent dietary habits and physical activity levels throughout the study. A wash–out period of 7 days was implemented between visits.

The study was conducted at Kyung Hee University Hospital (Seoul, Republic of Korea). All participants received detailed information regarding procedures and potential risks prior to providing written informed consent. The study followed the principles of the Declaration of Helsinki. All procedures involving human participants were approved by the Institutional Review Board of Kyung Hee University Hospital (KHUH 2024-04-017) and registered with the Clinical Research Information Service of the Korea Disease Control and Prevention Agency (Registration Number KCT0010119).

### 2.2. Participants

Thirty-three healthy adults were recruited through open announcements. To account for potential dropouts or ineligibility, five participants were initially included as backup participants. Ultimately, 27 participants met the eligibility criteria and were enrolled in the study. One participant withdrew consent and discontinued participation at the third visit. Inclusion criteria were as follows: adults aged 20 to 50 years, capable of consuming one to two bottles of Soju, with previous hangover experience, and categorized as normal or at-risk drinkers based on the AUDIT-K survey [18]. Exclusion criteria included body mass index (BMI) < 18.5 kg/m^2^ or ≥30.0 kg/m^2^; uncontrolled hypertension (systolic/diastolic blood pressure ≥ 160/100 mmHg); diabetes (diagnosed type 1 or 2 diabetes, fasting glucose ≥ 126 mg/dL, use of antidiabetic medication and insulin); renal or hepatic dysfunction (creatinine ≥ 1.5 mg/dL; AST or ALT ≥ 2.5 times the reference range); excessive alcohol consumption within one week prior to screening or planned excessive consumption during the study; typically consumes alcohol less than once per month or abstains; history or treatment of cardiovascular, gastrointestinal, endocrine-metabolic, hepatobiliary, psychiatric, alcohol or substance use disorders; renal, hematologic, or neoplastic disorders; use of enzyme-inducing or inhibiting drugs, aspirin, or medications associated with bleeding risk; blood donation within two months (whole blood) or one month (component blood) before the first visit, or transfusion within one month prior; known allergy or hypersensitivity to any investigational product ingredient; participation in another clinical study within one month; pregnancy or lactation; illiteracy or significant impairment in complying with the study protocol; and any individual deemed unsuitable for participation by the investigator.

### 2.3. Measurements

#### 2.3.1. Socio-Demographic and Anthropometric Measurements

During the screening visit, sociodemographic data were collected, including sex, date of birth, lifestyle, alcohol consumption, smoking status, and medical and family histories. Height and weight were measured at each visit while participants wore lightweight clothing. Height was assessed using a BSM370 (InBody Co., Ltd., Seoul, Republic of Korea) device, and weight was measured using an InBody 970 (InBody Co., Ltd., Seoul, Republic of Korea). All measurements were rounded to one decimal place.

#### 2.3.2. Analytical Method for Alcohol Profiles

Serum alcohol and acetaldehyde levels were assessed at each visit. After the blood was allowed to stand at 20–25 °C for 10–20 min, it was centrifuged at 3000 rpm for 15 min to separate serum and plasma. The separated samples were then refrigerated at 4–8 °C. Blood was collected in serum-separating tubes (SST) from venous draws. Serum alcohol concentrations were determined enzymatically using a Cobas c502 analyzer (Roche, New York, NY, USA), and serum acetaldehyde concentrations were analyzed using an acetaldehyde assay kit (Abcam, Cambridge, UK). Breath alcohol concentrations were measured at each visit after blood collection using a CA20FL expiratory alcohol measuring device (Caos Co., Daejeon, Republic of Korea). Measurements were recorded before and after alcohol ingestion at 0, 15, and 30 min, and at 1, 2, 4, 6, and 15 h.

#### 2.3.3. Hangover Symptom Questionnaire

Hangover symptoms were evaluated using a questionnaire developed based on the guidelines for assessing healthy functional foods for hangover cure [19]. The survey consisted of 18 items addressing headache, vomiting, fatigue, difficulty concentrating, thirst or dehydration, light sensitivity, sleep disturbances, excessive sweating, diarrhea, feelings of depression, sleepiness, dizziness, stomachache, nausea, muscle pain, heartburn, flushing of the body or face, and memory loss. Symptoms were scored across five levels: 1 (no symptoms), 2 (mild symptoms), 3 (moderate symptoms), 4 (severe symptoms), and 5 (very severe symptoms). Participants completed the questionnaire 15 h after alcohol intake.

#### 2.3.4. Safety Assessments

Laboratory test results and vital signs (including blood pressure, pulse rate, and temperature) were assessed before and after each visit. Blood pressure and pulse rate were measured in the sitting position using an automated blood pressure monitor FT-500 (Jawon Medical, Daejeon, Republic of Korea). Adverse events were monitored continuously through interviews during each visit.

#### 2.3.5. Dietary Assessments

Nutritional intake was evaluated using the 24-h dietary recall method. Based on earlier findings that meal composition affects alcohol hangovers [20], participants were instructed to maintain their habitual diets throughout the study. At each visit, participants reported dietary intake for that day, and a registered dietitian conducted interviews to document food choices. Nutrient intake was analyzed using CAN-Pro 6.0 (Korean Nutrition Society, Seoul, Republic of Korea).

#### 2.3.6. Physical Activity Assessments

Physical activity was evaluated using the International Physical Activity Questionnaire (IPAQ) [21]. IPAQ data were recorded weekly and reflected typical activity levels of participants. Activity levels were expressed as metabolic equivalent tasks (MET) per week, calculated as MET level × activity minutes per day × activity days per week. MET values were assigned based on intensity: 8 METs for vigorous activity, 4 METs for moderate activity, and 3.3 METs for walking.

### 2.4. Sample Size Calculation

Sample size calculations were conducted using G*Power version 3.1.9.7 (Heinrich-Heine-University Düsseldorf, Germany). A sample size of 20 participants was considered sufficient to detect differences in alcohol area under the curve (AUC), and five participants for acetaldehyde AUC, with 80% statistical power at a 5% α level, based on previous matched-pairs studies [22,23]. Consequently, a final sample size of 27 was estimated, accounting for a dropout rate of approximately 20%.

### 2.5. Statistical Analysis

Primary outcomes were changes in serum alcohol and acetaldehyde responses between each investigational product and placebo. Secondary outcomes included changes in breath alcohol levels and hangover symptom questionnaire scores.

All statistical analyses were performed using SAS version 9.4 (SAS Institute, Cary, NC, USA). Descriptive statistics were presented as means ± standard deviation (SD) for continuous variables, and as *n* (%) for categorical variables. AUCs for serum alcohol, serum acetaldehyde, and breath alcohol responses were calculated using the trapezoidal linear method from baseline to the 15-h endpoint. Maximum concentration (C_max_) and time to reach C_max_ (T_max_) were also determined. Corrected acetaldehyde AUCs were baseline-adjusted [24]. Group differences were analyzed using a linear mixed model (LMM). Pairwise comparisons were performed using Tukey adjustment, and adjusted *p*-values with 95% confidence intervals (CI) for mean differences were reported. All figures were generated using R version 4.4.1 and the ggplot2 package version 3.5.1 [25,26].

## 3. Results

### 3.1. General Characteristics, Physical Activity Level, and Daily Dietary Intake of Participants

General characteristics are presented in Table 1. The sex distribution was similar between groups. Mean age, height, weight, BMI, blood pressure, pulse rate, respiratory rate, and laboratory test results were all within the reference ranges. Results for physical activity level and daily dietary intake are presented in Table S1. No significant differences in these variables were observed between visits.

### 3.2. Alcohol Profiles

#### 3.2.1. Alcohol Concentration (AIM I: Ready Q vs. Placebo)

**Figure 1:**
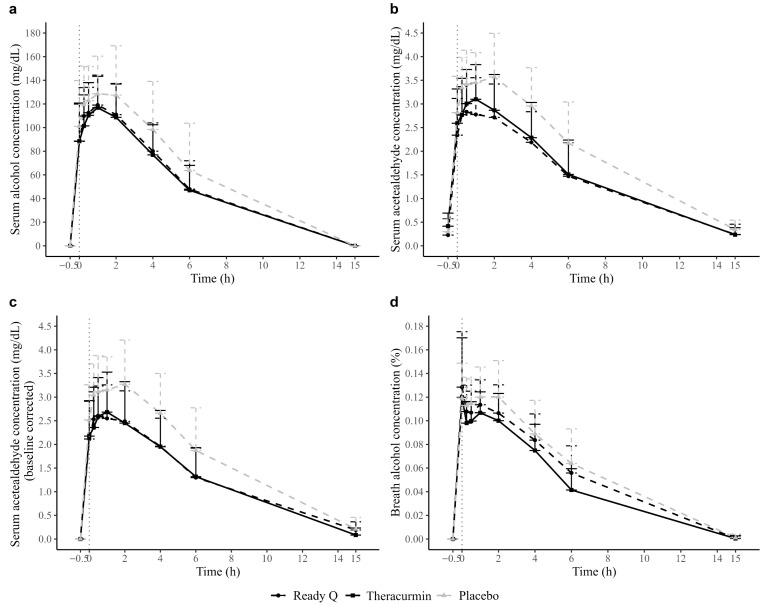
(**a**) Serum alcohol, (**b**) serum acetaldehyde, (**c**) corrected serum acetaldehyde, and (**d**) breath alcohol concentrations at each time point for Ready Q, Theracurmin, and placebo.

Serum alcohol concentrations at each time point, AUC, C_max_, and T_max_ are shown in Figure 1a, Table 2 and Table S2. Following alcohol consumption, Ready Q resulted in significantly lower serum alcohol concentrations compared to placebo at 2 h (−16.53 mg/dL [−30.14, −2.93]), 4 h (−18.36 mg/dL [−33.44, −3.27]), and 6 h (−15.81 mg/dL [−30.4, −1.23]). Serum alcohol AUC was significantly lower with Ready Q than with placebo (−94.92 mg·h/dL [−170.91, −18.93]). Similarly, serum alcohol C_max_ significantly decreased following Ready Q consumption compared to placebo (−17.60 mg/dL [−31.28, −3.92]).

Values are expressed as means ± SD or *n* (%). AUC, area under the curve; C_max_, maximum blood concentration; T_max_, time to reach C_max_; CI, confidence interval; The differences among the three groups were analyzed using a linear mixed model. Pairwise comparisons between groups were performed using Tukey adjustment, and the adjusted *p*-values along with the 95% confidence interval (CI) for the mean differences were calculated.

#### 3.2.2. Alcohol Concentration (AIM II: Theracurmin vs. Placebo)

Serum alcohol concentrations at each time point, AUC, C_max_, and T_max_ are shown in Figure 1a, Table 2 and Table S2. Theracurmin significantly reduced serum alcohol concentrations compared to placebo at 15 min (−17.76 mg/dL [−29.94, −5.59]), 30 min (−12.06 mg/dL [−22.47, −1.64]), 1 h (−11.58 mg/dL [−21.67, −1.49]), 2 h (−18.84 mg/dL [−32.63, −5.06]), 4 h (−21.79 mg/dL [−37.06, −6.52]), and 6 h (−17.40 mg/dL [−32.57, −2.23]). Serum alcohol AUC significantly decreased with Theracurmin compared to placebo (−117.21 mg·h/dL [−194.20, −40.22]), and C_max_ was also significantly lower (−22.97 mg/dL [−36.83, −9.11]).

#### 3.2.3. Acetaldehyde Concentration (AIM I: Ready Q vs. Placebo)

Serum acetaldehyde concentrations at each time point, along with AUC, C_max_, and T_max_, are shown in Figure 1b, Table 2 and Table S2. Ready Q led to significantly lower serum acetaldehyde concentrations than placebo at 0 min (−0.477 mg/dL [−0.845, −0.108]), 15 min (−0.582 mg/dL [−0.874, −0.290]), 30 min (−0.579 mg/dL [−0.899, −0.258]), 1 h (−0.675 mg/dL [−1.021, −0.330]), 2 h (−0.855 mg/dL [−1.227, −0.483]), 4 h (−0.771 mg/dL [−1.113, −0.429]), and 6 h (−0.700 mg/dL [−1.072, −0.327]). Serum acetaldehyde AUC significantly decreased with Ready Q compared to placebo (−8.441 mg·h/dL [−11.713, −5.169]), and C_max_ was also significantly lower (−0.833 mg/dL [−1.214, −0.453]).

#### 3.2.4. Acetaldehyde Concentration (AIM II: Theracurmin vs. Placebo)

Serum acetaldehyde concentrations at each time point, along with AUC, C_max_, and T_max_ values, are presented in Figure 1b, Table 2 and Table S2. Following alcohol drinking, Theracurmin resulted in significantly lower serum acetaldehyde concentrations compared to placebo at 15 min (−0.548 mg/dL [−0.844, −0.252]), 30 min (−0.386 mg/dL [−0.711, −0.062]), 2 h (−0.696 mg/dL [−1.073, −0.319]), 4 h (−0.673 mg/dL [−1.019, −0.326]), and 6 h (−0.670 mg/dL [−1.047, −0.292]). The serum acetaldehyde AUC was significantly lower with Theracurmin intake than with placebo (−7.080 mg·h/dL [−10.395, −3.765]). Similarly, serum acetaldehyde C_max_ significantly decreased following Theracurmin consumption compared to placebo (−0.621 mg/dL [−1.006, −0.235]).

#### 3.2.5. Corrected-Acetaldehyde Concentration (AIM I: Ready Q vs. Placebo)

Corrected serum acetaldehyde concentrations at each time point, along with AUC, C_max_, and T_max_, are shown in Figure 1c, Table 2 and Table S2. Following alcohol drinking, Ready Q resulted in significantly lower corrected serum acetaldehyde concentrations compared to placebo at 0 min (−0.405 mg/dL [−0.807, −0.002]), 15 min (−0.510 mg/dL [−0.841, −0.179]), 30 min (−0.507 mg/dL [−0.922, −0.091]), 1 h (−0.603 mg/dL [−1.010, −0.196]), 2 h (−0.783 mg/dL [−1.215, −0.351]), 4 h (−0.699 mg/dL [−1.101, −0.297]), and 6 h (−0.587 mg/dL [−0.997, −0.176]). The corrected serum acetaldehyde AUC significantly decreased with Ready Q intake compared to placebo (−6.895 mg·h/dL [−11.278, −2.512]). Additionally, corrected serum acetaldehyde C_max_ significantly decreased following Ready Q administration compared to placebo (−0.761 mg/dL [−1.211, −0.311]).

#### 3.2.6. Corrected Acetaldehyde Concentration (AIM II: Theracurmin vs. Placebo)

Corrected serum acetaldehyde concentrations at each time point, along with AUC, C_max_, and T_max_, are presented in Figure 1c, Table 2 and Table S2. Following alcohol drinking, Theracurmin led to significantly lower corrected serum acetaldehyde concentrations compared to placebo at 15 min (−0.662 mg/dL [−0.997, −0.327]), 30 min (−0.506 mg/dL [−0.926, −0.085]), 1 h (−0.460 mg/dL [−0.872, −0.049]), 2 h (−0.810 mg/dL [−1.248, −0.373]), 4 h (−0.721 mg/dL [−1.133, −0.310]), and 6 h (−0.571 mg/dL [−0.997, −0.144]). The corrected serum acetaldehyde AUC was significantly lower with Theracurmin compared to placebo (−8.161 mg·h/dL [−12.597, −3.725]). Similarly, corrected serum acetaldehyde C_max_ significantly decreased following Theracurmin administration compared to placebo (−0.737 mg/dL [−1.192, −0.282]).

#### 3.2.7. Breath Alcohol Concentration (AIM I: Ready Q vs. Placebo)

Breath alcohol concentrations at each time point and AUC values are presented in Figure 1d, Table 2 and Table S2. Following alcohol consumption, Ready Q led to significantly lower breath alcohol concentrations compared to placebo at 2 h (−0.014% [−0.024, −0.003]).

#### 3.2.8. Breath Alcohol Concentration (AIM II: Theracurmin vs. Placebo)

Breath alcohol concentrations at each time point and AUC values are shown in Figure 1d, Table 2 and Table S2. Following alcohol drinking, Theracurmin resulted in significantly lower breath alcohol concentrations compared to placebo at 15 min (−0.015% [−0.025, −0.005]), 30 min (−0.015% [−0.024, −0.006]), 1 h (−0.014% [−0.024, −0.004]), 2 h (−0.020% [−0.031, −0.010]), 4 h (−0.014% [−0.025, −0.003]), and 6 h (−0.023% [−0.035, −0.011]). The breath alcohol AUC significantly decreased following Theracurmin intake compared to placebo (−0.133%·h [−0.220, −0.046]).

#### 3.2.9. Hangover Symptoms (AIM I: Ready Q vs. Placebo)

Hangover symptoms are listed in Table S3. Among the 18 symptoms assessed, Ready Q significantly reduced the incidence of muscle pain (no symptoms: 100.00% vs. 70.37%; mild: 0.00% vs. 14.81%; moderate: 0.00% vs. 7.41%; severe: 0.00% vs. 3.70%; very severe: 0.00% vs. 3.70%; *p* = 0.0043) and flushing of the body or face (no symptoms: 81.48% vs. 66.67%; mild: 18.52% vs. 3.70%; moderate: 0.00% vs. 14.81%; severe: 0.00% vs. 7.41%; very severe: 0.00% vs. 7.41%; *p* = 0.0122) compared to placebo. The overall incidence of hangover symptoms was significantly lower with Ready Q than with placebo (no symptoms: 84.16% vs. 68.52%; mild: 11.93% vs. 12.35%; moderate: 3.09% vs. 10.08%; severe: 0.82% vs. 5.14%; very severe: 0.00% vs. 3.91%; *p* < 0.0001).

#### 3.2.10. Hangover Symptoms (AIM II: Theracurmin vs. Placebo)

Hangover symptoms are provided in Table S4. Among the 18 symptoms evaluated, Theracurmin significantly reduced the incidence of headache (no symptoms: 65.38% vs. 53.85%; mild: 34.62% vs. 15.38%; moderate: 0.00% vs. 11.54%; severe: 0.00% vs. 7.69%; very severe: 0.00% vs. 11.54%; *p* = 0.0248) and heartburn (no symptoms: 84.62% vs. 57.69%; mild: 15.38% vs. 15.38%; moderate: 0.00% vs. 11.54%; severe: 0.00% vs. 3.85%; very severe: 0.00% vs. 11.54%; *p* = 0.0473) compared to placebo. The overall incidence of hangover symptoms was significantly lower with Theracurmin than with placebo (no symptoms: 87.39% vs. 68.38%; mild: 11.11% vs. 12.18%; moderate: 1.28% vs. 10.04%; severe: 0.21% vs. 5.34%; very severe: 0.00% vs. 4.06%; *p* < 0.0001).

### 3.3. Safety Parameters

Laboratory test results and vital signs are summarized in Tables S5 and S6. Blood pressure, pulse rate, and temperature remained within reference ranges before and after each visit. One adverse event (vomiting) was reported in the placebo group, with full recovery observed before the visit concluded. No adverse events were deemed related to the investigational products, and no serious adverse events occurred.

## 4. Discussion

In this study, the effects of Ready Q and Theracurmin on alcohol metabolism profiles were evaluated in healthy adults and compared with those of a placebo. Ready Q and Theracurmin demonstrated reductions in serum alcohol AUC of 14.34% and 17.57%, respectively, relative to the placebo. Similarly, corrected acetaldehyde AUC decreased by 26.44% and 31.40%, respectively, with Ready Q and Theracurmin compared to the placebo. Notably, Ready Q significantly alleviated muscle pain and flushing of the body or face, while Theracurmin significantly reduced headache and heartburn. Moreover, the sum of hangover symptoms was significantly lower for both products than for the placebo. These results suggest that Ready Q and Theracurmin positively affect hangover symptoms.

Once alcohol is consumed, it is transported to the liver through the blood vessels in the small intestine. The primary organ for alcohol metabolism is the liver, which processes more than 90% of ethanol, and approximately 2–5% is excreted through urine, sweat, and breath without metabolism [27,28,29]. Alcohol transported to the liver is mainly oxidized to acetaldehyde by ADH through the alcohol dehydrogenase system. Additional pathways, including MEOS and the catalase system, contribute to alcohol metabolism [28,29,30]. These metabolisms produce acetaldehyde, a highly reactive and toxic substance oxidized by ALDH, which is subsequently converted into acetate. Acetate produced in the liver is released into the bloodstream and oxidized by peripheral tissues into carbon dioxide, fatty acids, and water [27,28]. As alcohol concentration decreases through these metabolic pathways, handling symptoms are caused by the toxicity of acetaldehyde and by-products [8]. To mitigate these symptoms, previous studies have focused on promoting alcohol metabolism in the liver to avoid acetaldehyde accumulation or using antioxidants to alleviate oxidative stress and hepatic toxicity arising from acetaldehyde. Anti-hangover drink with red ginseng reduces overall blood alcohol and results in fewer stomachaches, less thirst and dehydration, less difficulty concentrating, and less memory loss than a placebo [9]. Similarly, a complex containing four extracts known for alleviating hangover symptoms, such as fermented rice germ extracts and yeast extract mixtures, significantly reduced baseline-adjusted blood acetaldehyde AUC_6–12 h_ and improved thirst (15 h after drinking), shivering (1 h after drinking), and confusion (4 h after drinking) compared to a placebo [31]. Additionally, probiotics containing *Lactobacillus* and *Bifidobacterium* decreased blood alcohol and acetaldehyde after drinking for 6 h [32]. These findings provide valuable insights for developing more effective and specific hangover cures.

Theracurmin is a high-bioavailability form of curcumin, the antioxidant compound derived from turmeric [33]. Curcumin accelerates the activation of ALDH, facilitating the rapid elimination of acetaldehyde during alcohol metabolism [34]. As a result, the anti-hangover effects of Theracurmin have been confirmed in preclinical and clinical studies. Theracurmin and Theracurmin drinks (including *Hovenia dulcis*) enhance acetaldehyde clearance by reducing blood alcohol and acetaldehyde concentrations while boosting the activity of ADH and ALDH to accelerate alcohol metabolism in hangover-induced rat models [35]. Theracurmin drink lowers blood alcohol concentration more than the placebo [35]. In addition, 30 mg of Theracurmin significantly reduced blood acetaldehyde concentrations compared to mineral water while showing no significant effect on alcohol concentrations [16]. In our study, the consumption of 166.68 mg (containing 50 mg of curcumin), and 333.342 mg (containing 100 mg of curcumin) of Theracurmin led to a significant reduction in serum alcohol and acetaldehyde concentrations compared to the placebo. This was similar to previous results showing that 30 mg of Theracurmin led to a prominent reduction in acetaldehyde levels. These findings highlight the effect of Theracurmin on enhancing acetaldehyde clearance. Furthermore, curcumin exerts antioxidant and hepatoprotective effects by scavenging free radicals, inhibiting lipid peroxidation, modulating mitochondrial dysfunction, and restoring antioxidant enzyme activity, thereby providing potential protective effects in the liver [36].

Ready Q also contains yeast and *Hovenia dulcis* extracts, both of which are traditionally used as anti-hangover herbal medicines. Yeast extract, a complex mixture of amino acids, peptides, sugars, and other components, is particularly enriched with glutathione [37]. Glutathione, composed of cysteine, glycine, and glutamate, is a potent antioxidant that neutralizes reactive oxygen species, mitigates oxidative stress, and defends cells [38,39]. It also plays an important role in alcohol detoxification and is commonly utilized as a hangover cure [40]. Administration of yeast extract containing 50 mg glutathione significantly reduced blood acetaldehyde AUC_last_ by 51% and C_max_ by 53% compared to placebo [41]. In addition, *Hovenia dulcis* extract complexes commonly improved hangovers by rapidly reducing blood alcohol and acetaldehyde concentrations. A complex of *Hoveni dulcis* Thunb and Ginseng Berry extracts improved the hangover effect by reducing blood alcohol, blood acetaldehyde, and breath alcohol after drinking [42]. Furthermore, *Hoveni dulcis* Thunb extracts alone also decreased acetaldehyde, emphasizing its potential as an effective hangover cure [42]. Yeast extracts and *Hovenia dulcis* extracts significantly decreased plasma acetaldehyde AUC and C_max_ compared to placebo, respectively [23]. These results imply that the glutathione of yeast and *Hovenia dulcis* extracts are used as components to develop effective hangover cures.

No serious adverse events, abnormal vital signs, or laboratory test results were reported during our study, confirming the safety of Theracurmin CR-033P. A previous clinical trial showed that healthy individuals consuming 180 mg of curcumin for 2 or 12 months had no adverse effects on Theracurmin [43,44]. Additionally, consuming 900 mg of curcumin for 12 weeks had no adverse effect on Theracurmin [44]. Similar to previous studies, our safety outcome indicates that Theracurmin is safe for healthy adults to reduce hangover symptoms.

Although the clinical study report applied paired *t*-tests for comparisons between treatment conditions, LMM was applied in the present study to better account for repeated measures and individual variability. This analytic approach also accommodates unbalanced data owing to participant dropout and provides a more robust estimation of group effects over time [45]. In the present study, one participant withdrew before the final phase, resulting in incomplete data; the LMM framework allowed the inclusion of all available data under the assumption of missing at random. Notably, the pairwise comparisons derived from the LMM yielded results consistent with those from the original paired *t*-tests, indicating that the main findings remained robust, regardless of the statistical method applied (Tables S7 and S8).

This study has several strengths. First, we adopted a crossover design that ensured that each participant received both experimental and control interventions, thereby minimizing inter-individual variability and ensuring consistent intervention effects. Second, we recruited participants to ensure sex balance, thereby emphasizing the potential utility of our results in both male and female populations. Although previous studies predominantly recruited male participants [9,10,32,46,47,48,49,50,51], our study ensured an equal representation of both sexes. This balance allows for a more inclusive evaluation of the effects across both male and female populations, which are equally considered in the outcomes. However, this study has some limitations. The alcohol challenge dose (500 mL of Soju) was standardized based on the 2021 Korean Average Weight Based on General Health Screening (0.95 g alcohol/kg BW) without considering sex differences [52]. As a result, female participants, who generally have different body composition and hormone levels than males, may have experienced a relatively higher alcohol metabolism burden [53]. However, this approach was chosen to establish a standardized baseline for consistent comparisons across all participants. The focus of this study was to evaluate the effects of hangover treatments under controlled and consistent conditions. In addition, genetic polymorphisms of alcohol-metabolizing enzymes such as ADH and ALDH, particularly the ALDH2*2 variant that is prevalent in East Asian populations, were not assessed. This omission limits the ability to account for inter-individual differences in alcohol metabolism and hangover susceptibility. Future research should explore the potential influence of sex-specific variations in alcohol metabolism and consider dosage adjustments to enhance the applicability of these results. Finally, genetic screening of enzyme polymorphisms should be incorporated.

Therefore, this study demonstrated that the two hangover cures effectively improved the alcohol profiles of healthy adults. These results emphasize the potential of these products as tools for alleviating hangover symptoms after drinking.

## Figures and Tables

**Table 1:** General characteristics of the participants at baseline.

Variable		Total Participants (*n* = 27)	Male Participants (*n* = 14)	Female Participants (*n* = 13)
Age (y)		36.41 ± 8.30	35.64 ± 9.32	37.23 ± 7.33
Sex	Male	14 (52)	14 (100)	-
	Female	13 (48)	-	13 (100)
Height (cm)		169.67 ± 7.95	175.68 ± 4.90	162.79 ± 5.59
Weight (kg)		70.91 ± 11.59	78.50 ± 7.43	62.74 ± 9.57
BMI (kg/m^2^)		24.51 ± 2.80	25.30 ± 2.27	23.56 ± 3.17
Systolic blood pressure (mmHg)		125.59 ± 14.36	128.36 ± 15.37	122.62 ± 13.14
Diastolic blood pressure (mmHg)		79.93 ± 10.91	78.43 ± 10.70	81.54 ± 11.33
Pulse (beats/minute)		72.59 ± 11.24	68.29 ± 10.96	77.23 ± 9.93
Breathe (breaths/minute)		16.63 ± 2.87	16.64 ± 3.23	16.62 ± 2.57
Usual drinking alcohol (g/week)		156.90 ± 122.45	142.80 ± 75.86	172.08 ± 160.53
Smoking status	Yes	3 (11)	2 (14)	1 (8)
	No	19 (70)	7 (50)	12 (92)
	Ex	5 (19)	5 (36)	0 (0)
Sleep time	7 h or less	13 (48)	8 (57)	5 (39)
	7~8 h	11 (41)	5 (36)	6 (46)
	More than 8 h	3 (11)	1 (7)	2 (15)
Exercise	Yes	18 (67)	11 (79)	7 (54)
	No	9 (33)	3 (21)	6 (46)

Values are expressed as means ± SD or *n* (%). BMI, body mass index (weight(kg)/height(m)^2^).

**Table 2:** AUC, C_max_, and T_max_ of serum alcohol, serum acetaldehyde, corrected acetaldehyde, and breath alcohol for Ready Q, Theracurmin, and placebo.

Variable	Ready Q	Theracurmin	Placebo	Ready Q vs. Placebo	Theracurmin vs. Placebo
				Mean Difference (95% CI)	Mean Difference (95% CI)
**Alcohol**					
AUC (mg·h/dL)	567.06 ± 135.03	545.66 ± 153.06	661.99 ± 217.30	−94.92 (−170.91, −18.93)	−117.21 (−194.20, −40.22)
C_max_ (mg/dL)	127.39 ± 26.06	122.17 ± 27.82	144.99 ± 39.59	−17.60 (−31.28, −3.92)	−22.97 (−36.83, −9.11)
T_max_ (h)	1.39 ± 0.70	1.54 ± 0.67	1.46 ± 0.81	−0.07 (−0.41, 0.27)	0.06 (−0.28, 0.40)
**Acetaldehyde**					
AUC (mg·h/dL)	21.553 ± 8.021	22.922 ± 6.466	29.995 ± 8.000	−8.441 (−11.713, −5.169)	−7.080 (−10.395, −3.765)
C_max_ (mg/dL)	3.142 ± 0.701	3.346 ± 0.634	3.975 ± 0.866	−0.833 (−1.214, −0.453)	−0.621 (−1.006, −0.235)
T_max_ (h)	1.36 ± 0.66	1.46 ± 0.87	1.54 ± 0.80	−0.17 (−0.66, 0.32)	−0.08 (−0.57, 0.42)
**Corrected acetaldehyde**					
AUC (mg·h/dL)	19.187 ± 6.991	17.893 ± 7.725	26.082 ± 8.990	−6.895 (−11.278, −2.512)	−8.161 (−12.597, −3.725)
C_max_ (mg/dL)	2.914 ± 0.676	2.930 ± 0.742	3.675 ± 0.901	−0.761 (−1.211, −0.311)	−0.737 (−1.192, −0.282)
T_max_ (h)	1.36 ± 0.66	1.46 ± 0.87	1.54 ± 0.80	−0.17 (−0.66, 0.32)	−0.08 (−0.57, 0.42)
**Breath alcohol**					
AUC (%·h)	0.592 ± 0.122	0.535 ± 0.106	0.667 ± 0.233	−0.075 (−0.161, 0.011)	−0.133 (−0.220, −0.046)
C_max_ (%)	0.142 ± 0.038	0.132 ± 0.045	0.139 ± 0.028	0.003 (−0.013, 0.020)	−0.008 (−0.024, 0.009)
T_max_ (h)	1.13 ± 0.87	1.23 ± 0.81	1.33 ± 0.86	−0.20 (−0.61, 0.22)	−0.11 (−0.53, 0.31)

## Data Availability

The datasets generated during and/or analyzed during the current study are available from the corresponding author on reasonable request.

## Supplementary Materials

The following supporting information can be downloaded at https://www.mdpi.com/2077-0383/14/19/6996/s1: Table S1: Daily intake of energy and nutrients and physical activity for Ready Q, Theracurmin, and placebo; Table S2. Serum alcohol concentration, serum acetaldehyde concentration, corrected serum acetaldehyde concentration, and breath alcohol concentration at each time point for Ready Q, Theracurmin, and placebo; Table S3. Hangover symptoms after alcohol consumption for Ready Q and placebo; Table S4. Hangover symptoms after alcohol consumption for Theracurmin and placebo; Table S5. Laboratory tests of subjects at baseline for Ready Q, Theracurmin, and placebo; Table S6. Vital Signs of subjects for Ready Q, Theracurmin, and placebo; Table S7. Serum alcohol concentration, serum acetaldehyde concentration, corrected serum acetaldehyde concentration, and breath alcohol concentration at each time point for Ready Q and placebo as presented in the clinical study report; Table S8. Serum alcohol concentration, serum acetaldehyde concentration, corrected serum acetaldehyde concentration, and breath alcohol concentration at each time point for Theracurmin and placebo as presented in the clinical study report.

## Abbreviations

ADH	Alcohol Deghydrogenase
ALDH	Acetaldehyde Dehydrogenase
AUC	Areas Under the Curve
C_max_	Maximum Blood Concentration
T_max_	Time to reach C_max_
SD	Standard Deviation
BMI	Body Mass Index
