# Supplementary material for: Effect of Theracurmin Products for Alleviating Alcohol Hangovers in Healthy Adults
Source: J Clin Med. 2025 Oct 2;14(19):6996. doi: 10.3390/jcm14196996 (PMC12813635; doi:10.3390/jcm14196996)
Supplement: Supplementary file 1 [file jcm-14-06996-s001.zip › jcm-3846851-supplementary.pdf]

Table S1. Daily intake of energy and nutrients and physical activity for Ready Q, Theracurmin, and placebo

Variables	Ready Q	Theracurmin	Placebo	*P*-value[1)]	*P*-value[2)]
**Dietary intake**					
Energy (kcal)	1456.93 ± 427.42	1398.60 ± 317.89	1457.45 ± 352.08	0.6285[4)]	0.2184[3)]
Carbohydrate (g)	201.76 ± 42.75	201.76 ± 37.13	206.62 ± 46.88	0.5299[3)]	0.4346[3)]
Fat (g)	46.83 ± 28.26	41.80 ± 17.47	43.93 ± 19.89	0.9480[4)]	0.3885[4)]
Protein (g)	55.48 ± 22.10	51.34 ± 13.89	56.30 ± 20.99	0.9480[4)]	0.3168[4)]
Dietary fiber (g)	14.68 ± 5.14	13.56 ± 3.37	14.55 ± 4.68	0.3138[4)]	0.3885[4)]
Water (g)	555.39 ± 199.37	583.58 ± 225.02	602.52 ± 233.64	0.2387[4)]	0.0921[4)]
Vitamin A (ug RAE)	399.85 ± 123.00	341.44 ± 91.63	379.80 ± 113.07	0.2972[4)]	0.1014[4)]
Vitamin D (ug)	1.17 ± 0.96	1.07 ± 0.83	1.14 ± 0.89	0.8900[4)]	0.5171[4)]
Vitamin E (mg)	10.55 ± 4.06	9.85 ± 2.84	10.43 ± 3.51	0.6161[4)]	0.1635[3)]
Vitamin K (ug)	203.14 ± 75.85	208.75 ± 73.17	207.69 ± 69.50	0.2972[4)]	0.5459[4)]
Vitamin C (mg)	30.37 ± 16.01	31.30 ± 15.51	36.06 ± 18.48	0.1666[4)]	0.0973[4)]
Thiamine (mg)	0.99 ± 0.42	0.94 ± 0.44	1.01 ± 0.44	0.7048[4)]	0.4477[4)]
Riboflavin (mg)	1.25 ± 0.43	1.13 ± 0.30	1.22 ± 0.37	0.2728[4)]	0.1166[3)]
Niacin (mg)	14.28 ± 6.23	13.65 ± 3.12	13.78 ± 3.36	0.2728[4)]	0.7375[3)]
Vitamin B6 (mg)	0.53 ± 0.21	0.48 ± 0.27	0.48 ± 0.18	0.2317[4)]	0.3927[4)]
Folate (ug)	253.05 ± 90.49	216.55 ± 40.51	233.03 ± 55.47	0.0823[4)]	0.0511[4)]
Vitamin B12 (ug)	2.01 ± 1.32	1.90 ± 0.90	2.13 ± 1.23	0.6995[4)]	0.4180[4)]
Calcium (mg)	370.49 ± 107.58	358.95 ± 135.68	391.93 ± 157.32	0.7639[2)]	0.3698[2)]
Phosphorus (mg)	861.94 ± 297.72	800.02 ± 191.68	859.23 ± 238.74	0.8859[2)]	0.0664[2)]
Sodium (mg)	4247.68 ± 1233.81	3956.33 ± 718.59	4264.92 ± 1137.56	0.6472[2)]	0.0921[2)]
Potassium (mg)	2011.00 ± 488.07	1986.11 ± 384.75	2035.83 ± 542.72	0.8448[2)]	0.6261[2)]
Magnesium (mg)	157.65 ± 91.94	140.44 ± 45.60	150.93 ± 64.81	0.9065[2)]	0.1074[2)]
Iron (mg)	10.64 ± 3.36	10.17 ± 1.92	10.45 ± 2.44	0.9688[2)]	0.4077[2)]

Values are expressed as means ± SD.
1) *P*-values were obtained for Aim I: Comparison of Ready Q with Placebo.
2) *P*-values were obtained for Aim II: Comparison of Theracurmin with Placebo.
3) *P*-values were obtained by the Paired t-test, depending on the normality of the data.
4) *P*-values were obtained by the Wilcoxon signed-rank test, depending on the normality of the data.

Table S1. Daily intake of energy and nutrients and physical activity for Ready Q, Theracurmin, and placebo *(Continued)*

Variables	Ready Q	Theracurmin	Placebo	*P*-value[1)]	*P*-value[2)]
**Physical activity**					
Vigorous METs (METs-min/wk)	777.78 ± 1488.60	587.69 ± 1372.45	539.26 ± 1351.21	0.2500[4)]	0.2500[4)]
Moderate METs (METs-min/wk)	2883.74 ± 4023.59	2016.92 ± 2390.09	2551.11 ± 3284.54	0.8457[4)]	0.8457[4)]
Walking METs (METs-min/wk)	2429.78 ± 1257.71	2736.46 ± 1999.66	2506.78 ± 1937.67	1.0000[4)]	1.0000[4)]
Total METs (METs-min/wk)	6091.26 ± 4640.77	5341.08 ± 3524.58	5597.15 ± 4057.18	0.2100[4)]	0.2100[4)]

Values are expressed as means ± SD.

[1)] *P*-values were obtained for Aim I: Comparison of Ready Q with Placebo.

[2)] *P*-values were obtained for Aim II: Comparison of Theracurmin with Placebo.

[3)] *P*-values were obtained by the Paired t-test, depending on the normality of the data.

[4)] *P*-values were obtained by the Wilcoxon signed-rank test, depending on the normality of the data.

Table S2. Serum alcohol concentration, serum acetaldehyde concentration, corrected serum acetaldehyde concentration, and breath alcohol concentration at each time point for Ready Q, Theracurmin, and placebo

Variables	Ready Q	Theracurmin	Placebo	*P*-value[1)]	*P*-value[2)]
**Alcohol** (mg/dL)					
**Time points**					
-0.5h	0.00 ± 0.00	0.00 ± 0.00	0.00 ± 0.00	-	-
0h	88.38 ± 31.53	88.52 ± 32.18	101.11 ± 38.63	0.0948	0.1052
0.25h	109.82 ± 24.00	**101.44 ± 26.26**	119.48 ± 32.63	0.1381	**0.0026**
0.5h	112.61 ± 21.46	**110.38 ± 27.66**	122.82 ± 28.70	0.0519	**0.0196**
1h	119.03 ± 25.34	**116.80 ± 26.56**	128.55 ± 31.87	0.0638	**0.0209**
**2h**	**110.96 ± 26.34**	**108.71 ± 28.17**	127.50 ± 41.72	**0.0137**	**0.0050**
**4h**	**80.08 ± 23.92**	**76.96 ± 25.44**	98.43 ± 40.64	**0.0135**	**0.0033**
**6h**	**47.93 ± 24.03**	**47.01 ± 21.00**	63.74 ± 40.03	**0.0308**	**0.0211**
15h	0.00 ± 0.00	0.00 ± 0.00	0.00 ± 0.00	-	-
**AUC[3)]** (mg·h/dL)	**567.06 ± 135.03**	**545.66 ± 153.06**	661.99 ± 217.30	**0.0109**	**0.0016**
**C_{max}**	**127.39 ± 26.06**	**122.17 ± 27.82**	144.99 ± 39.59	**0.0086**	**0.0006**
**T_{max}**	1.39 ± 0.70	1.54 ± 0.67	1.46 ± 0.81	0.8637	0.8978
**Acetaldehyde** (mg/dL)					
**Time points**					
-0.5h	0.228 ± 0.347	0.416 ± 0.275	0.300 ± 0.262	0.6591	0.3464
0h	**2.340 ± 0.776**	2.593 ± 0.724	2.817 ± 0.767	**0.0081**	0.3282
**0.25h**	**2.762 ± 0.607**	**2.773 ± 0.769**	3.344 ± 0.642	**<0.0001**	**0.0001**
**0.5h**	**2.833 ± 0.581**	**3.007 ± 0.722**	3.411 ± 0.726	**0.0002**	**0.0160**
1h	**2.777 ± 0.777**	3.099 ± 0.733	3.453 ± 0.633	**<0.0001**	0.0537
**2h**	**2.716 ± 0.707**	**2.867 ± 0.756**	3.571 ± 0.922	**<0.0001**	**0.0001**
**4h**	**2.188 ± 0.648**	**2.287 ± 0.745**	2.959 ± 0.808	**<0.0001**	**<0.0001**
**6h**	**1.473 ± 0.708**	**1.510 ± 0.726**	2.172 ± 0.872	**0.0001**	**0.0002**
15h	0.241 ± 0.215	0.236 ± 0.148	0.344 ± 0.201	0.1354	0.1149
**AUC[3)]** (mg·h/dL)	**21.553 ± 8.021**	**22.922 ± 6.466**	29.995 ± 8.000	**<0.0001**	**<0.0001**
**C_{max}**	**3.142 ± 0.701**	**3.346 ± 0.634**	3.975 ± 0.866	**<0.0001**	**0.0008**
**T_{max}**	1.363 ± 0.657	1.463 ± 0.867	1.537 ± 0.799	0.6678	0.9238

AUC : area under the curve, C_{max} : maximum blood concentration, T_{max} : time to reach C_{max}

Values are expressed as means ± SD; Pairwise comparisons between groups were performed using Tukey adjustment

1) *P*-values were obtained for Aim I: Comparison of Ready Q with Placebo.

2) *P*-values were obtained for Aim II: Comparison of Theracurmin with Placebo.

3) Linear trapezoidal linear method

4) AUC was calculated based on reference[24]

Table S2. Serum alcohol concentration, serum acetaldehyde concentration, corrected serum acetaldehyde concentration, and breath alcohol concentration at each time point for Ready Q, Theracurmin, and placebo *(Continued)*

Variables	Ready Q	Theracurmin	Placebo	*P*-value[1)]	*P*-value[2)]
**Corrected-Acetaldehyde (mg/dL)**					
**Time points**					
-0.5h	0.000 ± 0.000	0.000 ± 0.000	0.000 ± 0.000	-	-
0h	**2.112 ± 0.802**	2.517 ± 0.743	2.177 ± 0.748	**0.0486**	0.1256
**0.25h**	**2.534 ± 0.578**	**3.044 ± 0.661**	2.357 ± 0.851	**0.0014**	**<0.0001**
**0.5h**	**2.605 ± 0.630**	**3.111 ± 0.767**	2.591 ± 0.822	**0.0134**	**0.0149**
**1h**	**2.549 ± 0.709**	**3.153 ± 0.699**	2.683 ± 0.845	**0.0022**	**0.0250**
**2h**	**2.488 ± 0.644**	**3.271 ± 0.936**	2.451 ± 0.875	**0.0002**	**0.0001**
**4h**	**1.960 ± 0.594**	**2.659 ± 0.841**	1.950 ± 0.767	**0.0003**	**0.0003**
**6h**	**1.298 ± 0.623**	**1.872 ± 0.901**	1.318 ± 0.613	**0.0033**	**0.0061**
15h	0.192 ± 0.172	0.210 ± 0.248	0.084 ± 0.149	0.8284	0.3470
**AUC[4)] (mg·h/dL)**	**19.187 ± 6.991**	**17.893 ± 7.725**	26.082 ± 8.990	**0.0011**	**0.0001**
**C_{max}**	**2.914 ± 0.676**	**2.930 ± 0.742**	3.675 ± 0.901	**0.0005**	**0.0008**
**T_{max}**	1.363 ± 0.657	1.463 ± 0.867	1.537 ± 0.799	0.6678	0.9238
**Breath alcohol (%)**					
**Time points**					
-0.5h	0.000 ± 0.000	0.000 ± 0.000	0.000 ± 0.000	-	-
0h	0.128 ± 0.047	0.120 ± 0.050	0.119 ± 0.029	0.5808	0.9983
0.25h	0.108 ± 0.022	**0.098 ± 0.018**	0.113 ± 0.024	0.4480	**0.0024**
0.5h	0.107 ± 0.018	**0.100 ± 0.016**	0.115 ± 0.021	0.1089	**0.0008**
1h	0.114 ± 0.021	**0.107 ± 0.018**	0.120 ± 0.025	0.2609	**0.0052**
**2h**	**0.106 ± 0.024**	**0.100 ± 0.023**	0.120 ± 0.031	**0.0088**	**0.0001**
4h	0.084 ± 0.022	**0.075 ± 0.022**	0.089 ± 0.028	0.4782	**0.0086**
6h	0.056 ± 0.023	**0.041 ± 0.018**	0.064 ± 0.029	0.2215	**<0.0001**
15h	0.000 ± 0.003	0.000 ± 0.002	0.001 ± 0.003	0.6214	0.5610
**AUC[3)] (%·h)**	0.592 ± 0.122	**0.535 ± 0.106**	0.667 ± 0.233	0.0983	**0.0015**
**C_{max}**	0.142 ± 0.038	0.132 ± 0.045	0.139 ± 0.028	0.8930	0.5234
**T_{max}**	1.130 ± 0.869	1.232 ± 0.814	1.325 ± 0.858	0.4906	0.7973

AUC : area under the curve, C_{max} : maximum blood concentration, T_{max} : time to reach C_{max}

Values are expressed as means ± SD; Pairwise comparisons between groups were performed using Tukey adjustment

[1)] *P*-values were obtained for Aim I: Comparison of Ready Q with Placebo.

[2)] *P*-values were obtained for Aim II: Comparison of Theracurmin with Placebo.

[3)] Linear trapezoidal linear method

[4)] AUC was calculated based on reference[24]

Table S3. Hangover symptoms after alcohol consumption for Ready Q and placebo

Variables	Ready Q	Placebo	*P*-value	Variables	Ready Q	Placebo	*P*-value
**Thirst or Dehydration**				**Nausea**			
No symptoms [1]	16 (59.26)[2]	13 (48.15)		No symptoms	26 (96.30)	20 (74.07)	
Mild symptoms	7 (25.93)	6 (22.22)		Mild symptoms	1 (3.70)	1 (3.70)	
Moderate symptoms	2 (7.41)	5 (18.52)	0.6855[4]	Moderate symptoms	0 (0.00)	2 (7.40)	0.0801[4]
Severe symptoms	2 (7.41)	2 (7.41)		Severe symptoms	0 (0.00)	3 (11.11)	
Very severe symptoms	0 (0.00)	1 (3.70)		Very severe symptoms	0 (0.00)	1 (3.70)	
**Sleepiness**				**Fatigue**			
No symptoms	13 (48.15)	13 (59.10)		No symptoms	22 (81.48)	18 (66.67)	
Mild symptoms	8 (29.63)	6 (27.30)		Mild symptoms	5 (18.52)	3 (11.11)	
Moderate symptoms	5 (18.52)	3 (13.60)	0.3695[4]	Moderate symptoms	0 (0.00)	2 (7.41)	0.1603[4]
Severe symptoms	1 (3.70)	0 (0.00)		Severe symptoms	0 (0.00)	2 (7.41)	
Very severe symptoms	0 (0.00)	0 (0.00)		Very severe symptoms	0 (0.00)	2 (7.41)	
**Headache**				**Stomachache**			
No symptoms	18 (66.67)	15 (55.55)		No symptoms	25 (92.59)	20 (74.07)	
Mild symptoms	7 (25.93)	4 (14.81)		Mild symptoms	2 (7.41)	4 (14.81)	
Moderate symptoms	2 (7.41)	3 (11.11)	0.2098[4]	Moderate symptoms	0 (0.00)	3 (11.11)	0.1122[4]
Severe symptoms	0 (0.00)	2 (7.41)		Severe symptoms	0 (0.00)	0 (0.00)	
Very severe symptoms	0 (0.00)	2 (11.11)		Very severe symptoms	0 (0.00)	0 (0.00)	
**Dizziness**				**Diarrhea**			
No symptoms	24 (88.89)	16 (59.26)		No symptoms	23 (85.19)	22 (81.48)	
Mild symptoms	3 (11.11)	5 (18.52)		Mild symptoms	3 (11.11)	3 (11.11)	
Moderate symptoms	0 (0.00)	3 (11.11)	0.0553[4]	Moderate symptoms	1 (3.70)	2 (7.41)	1.0000[4]
Severe symptoms	0 (0.00)	2 (7.41)		Severe symptoms	0 (0.00)	0 (0.00)	
Very severe symptoms	0 (0.00)	1 (3.70)		Very severe symptoms	0 (0.00)	0 (0.00)	
**Vomiting**				**Difficulty concentrating**			
No symptoms	24 (88.89)	21 (77.78)		No symptoms	24 (88.89)	19 (70.37)	
Mild symptoms	2 (7.41)	4 (14.81)		Mild symptoms	3 (11.11)	2 (7.41)	
Moderate symptoms	1 (3.70)	0 (0.00)	0.4150[4]	Moderate symptoms	0 (0.00)	4 (14.81)	0.1156[4]
Severe symptoms	0 (0.00)	1 (3.70)		Severe symptoms	0 (0.00)	1 (3.70)	
Very severe symptoms	0 (0.00)	1 (3.70)		Very severe symptoms	0 (0.00)	1 (3.70)	

[1] The scores covered 5 stages, including 1 (no symptoms), 2 (mild symptoms), 3 (moderate symptoms), 4 (severe symptoms), and 5 (very severe symptoms).
[2] n (%)
[3] *P*-values were obtained by the Chi-square test
[4] *P*-values were obtained by the Fisher's exact test.

Table S3. Hangover symptoms after alcohol consumption for Ready Q and placebo *(Continued)*

Variables	Ready Q	Placebo	*P*-value	Variables	Ready Q	Placebo	*P*-value
**Light sensitivity**				**Memory loss**			
No symptoms [1]	25 (95.59)[2]	21 (77.78)		No symptoms	24 (88.89)	23 (85.19)	
Mild symptoms	1 (3.70)	1 (3.70)		Mild symptoms	3 (11.11)	2 (7.41)	
Moderate symptoms	1 (3.70)	4 (14.81)	0.3410[4]	Moderate symptoms	0 (0.00)	1 (3.70)	1.0000[4]
Severe symptoms	0 (0.00)	1 (3.70)		Severe symptoms	0 (0.00)	1 (3.70)	
Very severe symptoms	0 (0.00)	0 (0.00)		Very severe symptoms	0 (0.00)	0 (0.00)	
**Sleep disturbances**				**Muscle pain**			
No symptoms	23 (85.19)	18 (66.67)		No symptoms	27 (100.00)	19 (70.37)	
Mild symptoms	3 (11.11)	3 (11.11)		Mild symptoms	0 (0.00)	4 (14.81)	
Moderate symptoms	1 (3.70)	3 (11.11)	0.3959[4]	Moderate symptoms	0 (0.00)	2 (7.41)	**0.0043**[4]
Severe symptoms	0 (0.00)	1 (3.70)		Severe symptoms	0 (0.00)	1 (3.70)	
Very severe symptoms	0 (0.00)	2 (7.41)		Very severe symptoms	0 (0.00)	1 (3.70)	
**Excessive sweating**				**Heartburn**			
No symptoms	25 (92.59)	13 (85.19)		No symptoms	21 (77.78)	15 (55.56)	
Mild symptoms	2 (7.41)	2 (7.41)		Mild symptoms	3 (11.11)	4 (14.81)	
Moderate symptoms	0 (0.00)	0 (0.00)	0.5468[4]	Moderate symptoms	2 (7.41)	4 (14.81)	0.3232[4]
Severe symptoms	0 (0.00)	2 (7.41)		Severe symptoms	1 (3.70)	1 (3.70)	
Very severe symptoms	0 (0.00)	0 (0.00)		Very severe symptoms	0 (0.00)	3 (11.11)	
**Feelings of depression**				**Flushing of the body or face**			
No symptoms	27 (100.00)	24 (88.89)		No symptoms	22 (81.48)	18 (66.67)	
Mild symptoms	0 (0.00)	1 (3.70)		Mild symptoms	5 (18.52)	1 (3.70)	
Moderate symptoms	0 (0.00)	2 (7.41)	0.2358[4]	Moderate symptoms	0 (0.00)	4 (14.81)	**0.0122**[4]
Severe symptoms	0 (0.00)	0 (0.00)		Severe symptoms	0 (0.00)	2 (7.41)	
Very severe symptoms	0 (0.00)	0 (0.00)		Very severe symptoms	0 (0.00)	2 (7.41)	
**Sum**							
No symptoms	409 (84.16)	333 (68.52)					
Mild symptoms	58 (11.93)	60 (12.35)					
Moderate symptoms	15 (3.09)	49 (10.08)	**<0.0001**[3]				
Severe symptoms	4 (0.82)	25 (5.14)					
Very severe symptoms	0 (0.00)	19 (3.91)					

[1] The scores covered 5 stages, including 1 (no symptoms), 2 (mild symptoms), 3 (moderate symptoms), 4 (severe symptoms), and 5 (very severe symptoms).
[2] n (%).
[3] *P*-values were obtained by the Chi-square test.
[4] *P*-values were obtained by the Fisher's exact test.

Table S4. Hangover symptoms after alcohol consumption for Theracurmin and placebo

Variables	Theracurmin	Placebo	*P*-value[3]	Variables	Theracurmin	Placebo	*P*-value
**Thirst or Dehydration**				**Nausea**			
No symptoms [1]	19 (73.08) [2]	13 (50.00)		No symptoms	24 (92.31)	20 (76.92)	
Mild symptoms	5 (19.23)	5 (19.23)		Mild symptoms	2 (7.69)	1 (3.85)	
Moderate symptoms	2 (7.69)	5 (19.23)	0.2443[4]	Moderate symptoms	0 (0.00)	1 (3.85)	0.2178[4]
Severe symptoms	0 (0.00)	2 (7.69)		Severe symptoms	0 (0.00)	3 (11.54)	
Very severe symptoms	0 (0.00)	1 (3.85)		Very severe symptoms	0 (0.00)	1 (3.85)	
**Sleepiness**				**Fatigue**			
No symptoms	11 (42.31)	8 (30.77)		No symptoms	21 (80.77)	17 (65.38)	
Mild symptoms	13 (50.00)	9 (34.62)		Mild symptoms	4 (15.38)	3 (11.54)	
Moderate symptoms	1 (3.85)	5 (19.23)	0.1529[4]	Moderate symptoms	1 (3.85)	2 (7.69)	0.3904[4]
Severe symptoms	1 (3.85)	4 (15.38)		Severe symptoms	0 (0.00)	2 (7.69)	
Very severe symptoms	0 (0.00)	0 (0.00)		Very severe symptoms	0 (0.00)	2 (7.69)	
**Headache**				**Stomachache**			
No symptoms	17 (65.38)	14 (53.85)		No symptoms	25 (96.15)	19 (73.08)	
Mild symptoms	9 (34.62)	4 (15.38)		Mild symptoms	1 (3.85)	4 (15.38)	
Moderate symptoms	0 (0.00)	3 (11.54)	**0.0248**[4]	Moderate symptoms	0 (0.00)	3 (11.54)	0.0790[4]
Severe symptoms	0 (0.00)	2 (7.69)		Severe symptoms	0 (0.00)	0 (0.00)	
Very severe symptoms	0 (0.00)	3 (11.54)		Very severe symptoms	0 (0.00)	0 (0.00)	
**Dizziness**				**Diarrhea**			
No symptoms	23 (88.46)	15 (57.69)		No symptoms	26 (100.00)	21 (80.77)	
Mild symptoms	3 (11.54)	5 (19.23)		Mild symptoms	0 (0.00)	3 (11.54)	
Moderate symptoms	0 (0.00)	3 (11.54)	0.0543[4]	Moderate symptoms	0 (0.00)	2 (7.69)	0.0506[4]
Severe symptoms	0 (0.00)	2 (7.699)		Severe symptoms	0 (0.00)	0 (0.00)	
Very severe symptoms	0 (0.00)	1 (3.85)		Very severe symptoms	0 (0.00)	0 (0.00)	
**Vomiting**				**Difficulty concentrating**			
No symptoms	26 (100.00)	21 (80.77)		No symptoms	24 (92.31)	18 (69.23)	
Mild symptoms	0 (0.00)	3 (11.54)		Mild symptoms	2 (7.69)	2 (7.69)	
Moderate symptoms	0 (0.00)	0 (0.00)	0.0506[4]	Moderate symptoms	0 (0.00)	4 (15.38)	0.1228[4]
Severe symptoms	0 (0.00)	1 (3.85)		Severe symptoms	0 (0.00)	1 (3.85)	
Very severe symptoms	0 (0.00)	1 (3.85)		Very severe symptoms	0 (0.00)	1 (3.85)	

[1] The scores covered 5 stages, including 1 (no symptoms), 2 (mild symptoms), 3 (moderate symptoms), 4 (severe symptoms), and 5 (very severe symptoms).
[2] n (%).
[3] *P*-values were obtained by the Chi-square test.
[4] *P*-values were obtained by the Fisher’s exact test.

Table S4. Hangover symptoms after alcohol consumption for Theracurmin and placebo *(Continued)*

Variables	Theracurmin	Placebo	*P*-value	Variables	Theracurmin	Placebo	*P*-value
**Light sensitivity**				**Memory loss**			
No symptoms [1]	25 (96.15)[2]	20 (76.92)		No symptoms	26 (100.00)	22 (84.62)	
Mild symptoms	0 (0.00)	1 (3.85)		Mild symptoms	0 (0.00)	2 (7.69)	
Moderate symptoms	1 (3.85)	4 (15.38)	0.1145[4]	Moderate symptoms	0 (0.00)	1 (3.85)	0.1104[4]
Severe symptoms	0 (0.00)	1 (3.85)		Severe symptoms	0 (0.00)	1 (3.85)	
Very severe symptoms	0 (0.00)	0 (0.00)		Very severe symptoms	0 (0.00)	0 (0.00)	
**Sleep disturbances**				**Muscle pain**			
No symptoms	21 (80.77)	17 (65.38)		No symptoms	25 (96.15)	18 (69.23)	
Mild symptoms	5 (19.23)	3 (11.54)		Mild symptoms	1 (3.85)	4 (15.38)	
Moderate symptoms	0 (0.00)	3 (11.54)	0.1145[4]	Moderate symptoms	0 (0.00)	2 (7.69)	0.0629[4]
Severe symptoms	0 (0.00)	1 (3.85)		Severe symptoms	0 (0.00)	1 (3.85)	
Very severe symptoms	0 (0.00)	2 (7.69)		Very severe symptoms	0 (0.00)	1 (3.85)	
**Excessive sweating**				**Heartburn**			
No symptoms	25 (96.15)	22 (84.62)		No symptoms	22 (84.62)	15 (57.69)	
Mild symptoms	1 (3.85)	2 (7.69)		Mild symptoms	4 (15.38)	4 (15.38)	
Moderate symptoms	0 (0.00)	0 (0.00)	0.4148[4]	Moderate symptoms	0 (0.00)	3 (11.54)	**0.0473**[4]
Severe symptoms	0 (0.00)	2 (7.69)		Severe symptoms	0 (0.00)	1 (3.85)	
Very severe symptoms	0 (0.00)	0 (0.00)		Very severe symptoms	0 (0.00)	3 (11.54)	
**Feelings of depression**				**Flushing of the body or face**			
No symptoms	26 (100.00)	23 (88.46)		No symptoms	23 (88.46)	17 (65.38)	
Mild symptoms	0 (0.00)	1 (3.85)		Mild symptoms	2 (7.69)	1 (3.85)	
Moderate symptoms	0 (0.00)	2 (7.69)	0.2353[4]	Moderate symptoms	1 (3.85)	4 (15.38)	0.1380[4]
Severe symptoms	0 (0.00)	0 (0.00)		Severe symptoms	0 (0.00)	2 (7.69)	
Very severe symptoms	0 (0.00)	0 (0.00)		Very severe symptoms	0 (0.00)	2 (7.69)	
**Sum**							
No symptoms	409 (87.39)	320 (68.38)					
Mild symptoms	52 (11.11)	57 (12.18)					
Moderate symptoms	6 (1.28)	47 (10.04)	**<0.0001**[3]				
Severe symptoms	1 (0.21)	25 (5.34)					
Very severe symptoms	0 (0.00)	19 (4.06)					

[1] The scores covered 5 stages, including 1 (no symptoms), 2 (mild symptoms), 3 (moderate symptoms), 4 (severe symptoms), and 5 (very severe symptoms).
[2] n (%).
[3] *P*-values were obtained by the Chi-square test.
[4] *P*-values were obtained by the Fisher's exact test.

Table S5. Laboratory tests of subjects at baseline for Ready Q, Theracurmin, and placebo

Variables	Reference Range	Ready Q	Theracurmin	Placebo
WBC (10^3/μL)	4.0 ~ 10.0 × 10^3/μL	6.17 ± 1.55	5.77 ± 1.41	5.85 ± 1.73
RBC (10^6/μL)	M: 4.2 ~ 6.3 × 10^6/μL<br>F: 4.0 ~ 5.4 × 10^6/μL	4.47 ± 0.39	4.42 ± 0.52	4.48 ± 0.42
Hb (g/dL)	M: 13 ~ 17 g/dL<br>F: 12 ~ 16 g/dL	13.83 ± 1.28	13.44 ± 1.46	13.54 ± 1.32
Hct (%)	M: 42 ~ 52 %<br>F: 37 ~ 47 %	41.57 ± 3.14	40.03 ± 3.95	41.58 ± 3.30
MCV (fL)	M: 80 ~ 94 fL<br>F: 81 ~ 99 fL	92.08 ± 2.82	91.69 ± 2.98	92.09 ± 2.89
MCH (pg)	27 ~ 31 pg	30.67 ± 0.81	30.59 ± 0.70	30.43 ± 0.79
MCHC (g/dL)	33 ~ 37 g/dL	33.34 ± 0.84	33.44 ± 0.76	33.06 ± 0.82
PLT (10^3/μL)	150 ~ 350 × 10^3/μL	248.11 ± 42.23	242.54 ± 38.20	252.22 ± 43.88
Total protein (g/dL)	6.6 ~ 8.3 g/dL	6.88 ± 0.33	6.77 ± 0.45	6.84 ± 0.35
Albumin (g/dL)	3.5 ~ 5.2 g/dL	4.39 ± 0.22	4.41 ± 0.26	4.40 ± 0.21
BUN (mg/dL)	8 ~ 20 mg/dL	12.30 ± 2.55	12.31 ± 2.46	12.52 ± 2.78
Cr (mg/dL)	M : 0.67 ~ 1.17 mg/dL<br>F : 0.51 ~ 0.95 mg/dL	0.81 ± 0.21	0.80 ± 0.19	0.84 ± 0.20
AST (U/L)	M : < 50 U/L<br>F : < 35 U/L	25.00 ± 15.01	21.15 ± 5.16	21.07 ± 6.37
ALT (U/L)	M : < 50 U/L<br>F : < 35 U/L	21.48 ± 16.56	17.12 ± 11.89	20.67 ± 18.62
Glucose (mg/dL)	74 ~ 106 mg/dL	86.70 ± 5.75	86.31 ± 6.71	87.70 ± 9.22
pH	5.0 ~ 8.0	5.97 ± 0.61	5.79 ± 0.47	6.03 ± 0.51
Nitrite	Negative			
Negative		27 (100)	27 (100)	27 (100)
S.G	1.010 ~ 1.025	1.02 ± 0.01	1.02 ± 0.01	1.02 ± 0.00
Protein	≤ Trace			
Negative		22 (81)	20 (74)	23 (85)
Trace		4 (15)	7 (26)	4 (15)
1 Positive		1 (4)	0 (0)	0 (0)
Glucose	Negative			
Negative		27 (100)	27 (100)	27 (100)
Ketone	Negative			
Negative		26 (96)	27 (100)	27 (100)
Trace		1 (4)	0 (0)	0 (0)
Bilirubin	Negative			
Negative		27 (100)	27 (100)	27 (100)
Occult Blood	Negative			
Negative		22 (82)	21 (77)	20 (74)
Trace		1 (4)	4 (15)	3 (11)
1 Positive		2 (7)	0 (0)	1 (4)
2 Positive		0 (0)	1 (4)	0 (0)
3 Positive		2 (7)	1 (4)	3 (11)
Urobilinogen	±			
±		27 (100)	27 (100)	27 (100)
Leukocyte esterase	Negative			
Negative		24 (88)	21 (77)	22 (81)
Trace		1 (4)	1 (4)	4 (15)
1 Positive		1 (4)	3 (11)	1 (4)
2 Positive		0 (0)	1 (4)	0 (0)
3 Positive		1 (4)	1 (4)	0 (0)

Values are expressed as means ± SD or n (%)

Table S6. Vital Signs of subjects for Ready Q, Theracurmin, and placebo

Variables	Ready Q	Theracurmin	Placebo
**Systolic blood pressure (mmHg)**			
Pre	127.48 ± 16.57	122.31 ± 12.98	124.58 ± 12.51
Post	121.78 ± 12.46	120.71 ± 8.58	121.25 ± 11.95
**Diastolic blood pressure (mmHg)**			
Pre	92.08 ± 2.82	72.27 ± 8.86	74.33 ± 10.00
Post	30.67 ± 0.81	70.32 ± 8.78	72.67 ± 8.61
**Pulse (beats/minute)**			
Pre	75.41 ± 11.22	79.08 ± 11.47	79.79 ± 12.64
Post	70.22 ± 10.24	69.52 ± 10.59	71.08 ± 9.56
**Breathe (breaths/minute)**			
Pre	17.78 ± 2.41	19.46 ± 0.51	19.21 ± 0.72
Post	18.52 ± 2.23	18.96 ± 0.60	18.25 ± 2.79
**Temperature (℃)**			
Pre	36.75 ± 0.27	36.76 ± 0.30	36.82 ± 0.30
Post	36.60 ± 0.27	36.54 ± 0.33	36.79 ± 0.30

Values are expressed as means ± SD.

Table S7. Serum alcohol concentration, serum acetaldehyde concentration, corrected serum acetaldehyde concentration, and breath alcohol concentration at each time point for Ready Q and placebo as presented in the clinical study report

Variables	Ready Q	Placebo	Change	*P*-value[1)]
**Alcohol** **(mg/dL)**				
**Time points**				
-0.5h	0.00 ± 0.00	0.00 ± 0.00	0.00 ± 0.00	-
0h	88.38 ± 31.53	101.11 ± 38.63	12.73 ± 35.33	0.0725[3)]
0.25h	109.82 ± 24.00	119.48 ± 32.63	9.66 ± 27.34	0.0778[3)]
**0.5h**	**112.61 ± 21.46**	**122.82 ± 28.70**	**10.21 ± 20.72**	**0.0166[3)]**
**1h**	**119.03 ± 25.34**	**128.55 ± 31.87**	**9.52 ± 22.26**	**0.0352[3)]**
**2h**	**110.96 ± 26.34**	**127.50 ± 41.72**	**16.53 ± 32.75**	**0.0011[4)]**
**4h**	**80.08 ± 23.92**	**98.43 ± 40.64**	**18.36 ± 35.78**	**0.0006[4)]**
**6h**	**47.93 ± 24.03**	**63.74 ± 40.03**	**15.81 ± 35.82**	**0.0296[4)]**
15h	0.00 ± 0.00	0.00 ± 0.00	0.00 ± 0.00	-
**AUC[2)] (mg·h/dL)**	**567.06 ± 135.03**	**661.99 ± 217.30**	**94.92 ± 177.83**	**0.0101[3)]**
**C_{max}**	**127.39 ± 26.06**	**144.99 ± 39.59**	**17.60 ± 32.22**	**0.0087[3)]**
T_{max}	1.39 ± 0.70	1.46 ± 0.81	0.07 ± 0.78	0.8498[4)]
**Acetaldehyde** **(mg/dL)**				
**Time points**				
-0.5h	0.228 ± 0.347	0.300 ± 0.262	0.072 ± 0.429	0.1266[4)]
**0h**	**2.340 ± 0.776**	**2.817 ± 0.767**	**0.477 ± 0.808**	**0.0033[4)]**
**0.25h**	**2.762 ± 0.607**	**3.344 ± 0.642**	**0.582 ± 0.540**	**<0.0001[3)]**
**0.5h**	**2.833 ± 0.581**	**3.411 ± 0.726**	**0.579 ± 0.704**	**0.0002[3)]**
**1h**	**2.777 ± 0.777**	**3.453 ± 0.633**	**0.675 ± 0.775**	**0.0001[3)]**
**2h**	**2.716 ± 0.707**	**3.571 ± 0.922**	**0.855 ± 0.851**	**<0.0001[4)]**
**4h**	**2.188 ± 0.648**	**2.959 ± 0.808**	**0.771 ± 0.774**	**<0.0001[3)]**
**6h**	**1.473 ± 0.708**	**2.172 ± 0.872**	**0.700 ± 0.830**	**0.0002[3)]**
15h	0.241 ± 0.215	0.344 ± 0.201	0.103 ± 0.301	0.0594[4)]
**AUC[2)] (mg·h/dL)**	**21.553 ± 8.021**	**29.995 ± 8.000**	**8.441 ± 7.119**	**<0.0001[3)]**
**C_{max}**	**3.142 ± 0.701**	**3.975 ± 0.866**	**0.833 ± 0.867**	**<0.0001[3)]**
T_{max}	1.363 ± 0.657	1.537 ± 0.799	0.174 ± 0.950	0.4462[4)]

AUC : area under the curve, C_{max} : maximum blood concentration, T_{max} : time to reach C_{max}

Values are expressed as means ± SD.

1) *P*-values were obtained for Aim I: Comparison of Ready Q with Placebo.

2) Linear trapezoidal linear method

3) *P*-values were obtained using the Paired t-test, depending on the normality of the data.

4) *P*-values were obtained using the Wilcoxon signed-rank test, depending on the normality of the data.

5) AUC was calculated based on reference[24]

Table S7. Serum alcohol concentration, serum acetaldehyde concentration, corrected serum acetaldehyde concentration, and breath alcohol concentration at each time point for Ready Q and placebo as presented in the clinical study report *(Continued)*

Variables	Ready Q	Placebo	Change	*P*-value[1)]
**Corrected-Acetaldehyde (mg/dL)**				
**Time points**				
-0.5h	0.000 ± 0.000	0.000 ± 0.000	0.000 ± 0.000	-
**0h**	**2.112 ± 0.802**	**2.517 ± 0.743**	**0.405 ± 0.910**	**0.0240**[4)]
**0.25h**	**2.534 ± 0.578**	**3.044 ± 0.661**	**0.510 ± 0.673**	**0.0003**[4)]
**0.5h**	**2.605 ± 0.630**	**3.111 ± 0.767**	**0.506 ± 0.958**	**0.0107**[3)]
**1h**	**2.549 ± 0.709**	**3.153 ± 0.699**	**0.603 ± 0.872**	**0.0013**[3)]
**2h**	**2.488 ± 0.644**	**3.271 ± 0.936**	**0.783 ± 0.979**	**<0.0001**[4)]
**4h**	**1.960 ± 0.594**	**2.659 ± 0.841**	**0.699 ± 0.893**	**0.0004**[3)]
**6h**	**1.298 ± 0.623**	**1.872 ± 0.901**	**0.616 ± 0.989**	**0.0039**[3)]
15h	0.192 ± 0.172	0.210 ± 0.248	-0.070 ± 0.327	0.5117[4)]
**AUC[5)] (mg·h/dL)**	**19.187 ± 6.991**	**26.082 ± 8.990**	**6.895 ± 10.133**	**0.0015**[3)]
**C_{max}**	**2.914 ± 0.676**	**3.675 ± 0.901**	**0.761 ± 1.065**	**0.0001**[4)]
T_{max}	1.363 ± 0.657	1.537 ± 0.799	0.174 ± 0.950	0.4462[4)]
**Breath alcohol (%)**				
**Time points**				
-0.5h	0.000 ± 0.000	0.000 ± 0.000	0.000 ± 0.000	-
0h	0.128 ± 0.047	0.119 ± 0.029	-0.009 ± 0.048	0.3313[3)]
0.25h	0.108 ± 0.022	0.113 ± 0.024	0.005 ± 0.024	0.2895[3)]
0.5h	0.107 ± 0.018	0.115 ± 0.021	0.008 ± 0.023	0.0892[3)]
1h	0.114 ± 0.021	0.120 ± 0.025	0.007 ± 0.023	0.1582[3)]
**2h**	**0.106 ± 0.024**	0.120 ± 0.031	**0.014 ± 0.027**	**0.0125**[3)]
4h	0.084 ± 0.022	0.089 ± 0.028	0.005 ± 0.026	0.6273[4)]
6h	0.056 ± 0.023	0.064 ± 0.029	0.008 ± 0.027	0.3584[4)]
15h	0.000 ± 0.003	0.001 ± 0.003	0.001 ± 0.004	0.8750[4)]
AUC[2)] (%·h)	0.592 ± 0.122	0.667 ± 0.233	0.075 ± 0.224	0.1032[4)]

AUC : area under the curve, C_{max} : maximum blood concentration, T_{max} : time to reach C_{max}

Values are expressed as means ± SD.

[1)] *P*-values were obtained for Aim I: Comparison of Ready Q with Placebo.

[2)] Linear trapezoidal linear method

[3)] *P*-values were obtained using the Paired t-test, depending on the normality of the data.

[4)] *P*-values were obtained using the Wilcoxon signed-rank test, depending on the normality of the data.

[5)] AUC was calculated based on reference[24]

Table S8. Serum alcohol concentration, serum acetaldehyde concentration, corrected serum acetaldehyde concentration, and breath alcohol concentration at each time point for Theracurmin and placebo as presented in the clinical study report

Variables	Theracurmin	Placebo	Change	*P*-value[1)]
**Alcohol** (mg/dL)				
**Time points**				
-0.5h	0.00 ± 0.00	0.00 ± 0.00	0.00 ± 0.00	-
0h	88.52 ± 32.18	100.47 ± 39.25	11.94 ± 28.58	0.0816[4)]
**0.25h**	**101.44 ± 26.26**	**118.80 ± 33.08**	**17.36 ± 31.27**	**0.0086[4)]**
**0.5h**	**110.38 ± 27.66**	**122.31 ± 29.15**	**11.92 ± 24.75**	**0.0332[4)]**
**1h**	**116.80 ± 26.56**	**128.25 ± 32.46**	**11.45 ± 25.40**	**0.0302[3)]**
**2h**	**108.71 ± 28.17**	**127.59 ± 42.54**	**18.88 ± 37.07**	**0.0004[4)]**
**4h**	**76.96 ± 25.44**	**99.05 ± 41.32**	**22.10 ± 42.35**	**0.0005[4)]**
**6h**	**47.01 ± 21.00**	**64.70 ± 40.51**	**15.68 ± 33.98**	**0.0117[4)]**
15h	0.00 ± 0.00	0.00 ± 0.00	0.00 ± 0.00	-
**AUC[2)] (mg·h/dL)**	**545.66 ± 153.06**	**663.33 ± 221.49**	**117.68 ± 210.39**	**0.0086[3)]**
**C_{max}**	**122.17 ± 27.82**	**145.29 ± 40.34**	**23.12 ± 35.74**	**0.0029[3)]**
T_{max}	1.54 ± 0.67	1.49 ± 0.81	-0.05 ± 0.75	0.9137[4)]
**Acetaldehyde** (mg/dL)				
**Time points**				
**-0.5h**	**0.416 ± 0.275**	**0.306 ± 0.266**	**-0.111 ± 0.405**	**0.0026[4)]**
0h	2.593 ± 0.724	2.815 ± 0.783	0.222 ± 0.770	0.1536[3)]
**0.25h**	**2.773 ± 0.769**	**3.314 ± 0.636**	**0.541 ± 0.761**	**0.0008[4)]**
**0.5h**	**3.007 ± 0.722**	**3.393 ± 0.734**	**0.386 ± 0.789**	**0.0183[4)]**
**1h**	**3.099 ± 0.733**	**3.460 ± 0.644**	**0.361 ± 0.803**	**0.0356[4)]**
**2h**	**2.867 ± 0.756**	**3.574 ± 0.941**	**0.707 ± 0.983**	**0.0001[4)]**
**4h**	**2.287 ± 0.745**	**2.968 ± 0.823**	**0.681 ± 0.882**	**0.0001[4)]**
**6h**	**1.510 ± 0.726**	**2.197 ± 0.879**	**0.687 ± 0.997**	**0.0017[3)]**
**15h**	**0.236 ± 0.148**	**0.355 ± 0.197**	**0.119 ± 0.272**	**0.0029[4)]**
**AUC[2)] (mg·h/dL)**	**22.922 ± 6.466**	**30.167 ± 8.107**	**7.245 ± 7.759**	**<0.0001[4)]**
**C_{max}**	**3.346 ± 0.634**	**3.970 ± 0.883**	**0.624 ± 0.963**	**0.0005[4)]**
T_{max}	1.463 ± 0.867	1.567 ± 0.799	0.104 ± 1.209	0.3725[4)]

AUC : area under the curve, C_{max} : maximum blood concentration, T_{max} : time to reach C_{max}
Values are expressed as means ± SD.
1) *P*-values were obtained for Aim II: Comparison of Theracurmin with Placebo.
2) Linear trapezoidal linear method
3) *P*-values were obtained using the Paired t-test, depending on the normality of the data.
4) *P*-values were obtained using the Wilcoxon signed-rank test, depending on the normality of the data.
5) AUC was calculated based on reference[24]

Table S8. Serum alcohol concentration, serum acetaldehyde concentration, corrected serum acetaldehyde concentration, and breath alcohol concentration at each time point for Theracurmin and placebo as presented in the clinical study report *(Continued)*

Variables	Theracurmin	Placebo	Change	*P*-value[1)]
**Corrected-Acetaldehyde (mg/dL)**				
**Time points**				
-0.5h	0.000 ± 0.000	0.000 ± 0.000	0.000 ± 0.000	-
**0h**	**2.177 ± 0.748**	**2.510 ± 0.757**	**0.333 ± 0.797**	**0.0356[4)]**
**0.25h**	**2.357 ± 0.851**	**3.009 ± 0.648**	**0.652 ± 0.798**	**0.0003[3)]**
**0.5h**	**2.591 ± 0.822**	**3.087 ± 0.772**	**0.496 ± 0.901**	**0.0095[3)]**
**1h**	**2.683 ± 0.845**	**3.155 ± 0.713**	**0.472 ± 0.956**	**0.0186[3)]**
**2h**	**2.451 ± 0.875**	**3.269 ± 0.955**	**0.818 ± 1.092**	**0.0002[4)]**
**4h**	**1.950 ± 0.767**	**2.662 ± 0.858**	**0.747 ± 0.982**	**0.0009[3)]**
**6h**	**1.318 ± 0.613**	**1.892 ± 0.913**	**0.577 ± 0.904**	**0.0057[3)]**
15h	0.084 ± 0.149	0.210 ± 0.248	0.052 ± 0.336	0.8750[4)]
**AUC[5)] (mg·h/dL)**	**17.893 ± 7.725**	**26.197 ± 9.148**	**8.304 ± 10.380**	**0.0004[3)]**
**C_{max}**	**2.930 ± 0.742**	**3.664 ± 0.918**	**0.735 ± 1.055**	**0.0002[4)]**
T_{max}	1.463 ± 0.867	1.567 ± 0.799	0.104 ± 1.209	0.3725[4)]
**Breath alcohol (%)**				
**Time points**				
-0.5h	0.000 ± 0.000	0.000 ± 0.000	0.000 ± 0.000	-
0h	0.120 ± 0.050	0.119 ± 0.030	0.000 ± 0.046	0.0993[3)]
**0.25h**	**0.098 ± 0.018**	**0.113 ± 0.024**	**0.015 ± 0.019**	**0.0004[3)]**
**0.5h**	**0.100 ± 0.016**	**0.115 ± 0.021**	**0.016 ± 0.017**	**0.0001[4)]**
**1h**	**0.107 ± 0.018**	**0.120 ± 0.026**	**0.014 ± 0.020**	**0.0016[3)]**
**2h**	**0.100 ± 0.023**	**0.121 ± 0.031**	**0.021 ± 0.027**	**0.0007[3)]**
**4h**	**0.075 ± 0.022**	**0.089 ± 0.029**	**0.014 ± 0.030**	**0.0089[4)]**
**6h**	**0.041 ± 0.018**	**0.065 ± 0.030**	**0.023 ± 0.030**	**<0.0001[4)]**
15h	0.000 ± 0.002	0.001 ± 0.003	0.001 ± 0.004	0.8750[4)]
**AUC[2)] (%·h)**	**0.535 ± 0.106**	**0.671 ± 0.236**	**0.136 ± 0.222**	**<0.0001[4)]**

AUC : area under the curve, C_{max} : maximum blood concentration, T_{max} : time to reach C_{max}

Values are expressed as means ± SD.

[1)] *P*-values were obtained for Aim II: Comparison of Theracurmin with Placebo.

[2)] Linear trapezoidal linear method

[3)] *P*-values were obtained using the Paired t-test, depending on the normality of the data.

[4)] *P*-values were obtained using the Wilcoxon signed-rank test, depending on the normality of the data.

[5)] AUC was calculated based on reference[24]
